# A droplet microfluidic strategy for cultivation, investigation, and high-throughput isolation of mouse gut microbiome bacteria

**DOI:** 10.1128/aem.00695-25

**Published:** 2025-07-10

**Authors:** Sundar Hengoju, Ketema Abdissa, Santiago T. Boto, Ashkan Samimi, Karin Martin, Ilse D. Jacobsen, Miriam A. Rosenbaum

**Affiliations:** 1 Leibniz Institute for Natural Product Research and Infection Biology-Hans-Knöll-Institute 28406, Jena, Germany; 2 Faculty of Biological Sciences, Friedrich Schiller University Jena 9378, Jena, Germany; 3 Cluster of Excellence Balance of the Microverse, Friedrich Schiller University 9378, Jena, Germany; Kyoto University, Kyoto, Japan

**Keywords:** droplet microfluidics, microbial cultivation, mouse gut microbiota, bacterial diversity

## Abstract

Understanding the gut microbiome’s intricate dynamics and its impact on host health necessitates the cultivation and isolation of its constituent microorganisms. Traditional culturing techniques often fall short in capturing the diversity of the gut microbiota, particularly for rare and slow-growing species. In this study, we present a droplet microfluidic platform as a high-throughput and efficient method for the cultivation and isolation of mouse gut microorganisms. Droplets, each encapsulating a single cell, were incubated under both aerobic and anaerobic conditions, thereby providing individual microenvironments without nutrient competition and facilitating the growth of a wide range of microorganisms. We validated the platform by successfully cultivating and isolating a diverse array of gut microorganisms, including strains with probiotic potential. A comparative analysis with traditional agar plating techniques revealed a higher number of unique isolates from the droplet cultivation method, demonstrating its enhanced capability to capture the cultivable fraction of the gut microbiome. Beyond isolation, 16S rDNA amplicon sequencing of the diverse microbial cultures in droplets demonstrated that our system reflects changes in microbial diversity induced by dietary interventions in mice. Droplet microfluidics offers a powerful and scalable tool for the high-throughput cultivation, evaluation, and isolation of gut microorganisms, paving the way for deeper insights into the gut microbiome’s role in health and disease.

**IMPORTANCE:**

The gut microbiome plays a crucial role in health and disease, yet many of its microbial members remain difficult to cultivate using traditional methods. In this study, we present a droplet microfluidic platform that advances our ability to cultivate, isolate, and analyze mouse gut microorganisms. By providing individual microenvironments for single cells, this high-throughput method overcomes limitations of traditional culturing techniques, enhancing microbial diversity recovery compared to standard techniques. Furthermore, this platform can reflect changes in microbial diversity in response to dietary changes in mice, highlighting its potential for studying gut microbial dynamics.

## INTRODUCTION

The human and animal gut microbiome represents an intricate ecosystem harboring trillions of microorganisms. This complex microbial community plays a crucial role in various aspects of human health and disease, influencing processes like metabolism and immune functions (1, 2). Dysbiosis, or an imbalance in the gut microbiota composition, has been implicated in a wide array of disorders, including inflammatory bowel diseases, metabolic disorders, obesity, diabetes, neurological conditions, and even certain cancers (3–6). Unraveling the intricate interplay between the gut microbiome and the host physiology is paramount for advancing our understanding of these multifaceted relationships and paving the way for novel therapeutic interventions (7, 8).

Recent advancements in high-throughput sequencing technologies have revolutionized our understanding of the taxonomic composition of the gut microbiome, enabling rapid community profiling and shedding light on the vast diversity of microorganisms, including many unculturable members. While these culture-independent approaches have significantly expanded our knowledge of the gut microbiome’s taxonomic landscape and their correlations with host health and disease states, they are limited in elucidating the physiological functions, metabolic capabilities, and interspecies interactions that govern the microbial ecology (9–13). To unravel these complexities, the isolation and cultivation of individual microbial strains remain indispensable. Traditional culture-based methods, such as agar plate techniques, have been instrumental in this endeavor, yet they are often limited in their ability to capture the full breadth of microbial diversity present in the gut (14–17). These conventional approaches are not only labor-intensive and low-throughput but also show bias toward cultivable taxa.

Droplet microfluidics, an emerging technology that harnesses the power of miniaturization, compartmentalization, and parallelization, provides a new strategy for microbial cultivations (18–22). This innovative approach enables the encapsulation of single cells or small consortia of microorganisms into picoliter to nanoliter-sized droplets, effectively transforming each droplet into a miniature bioreactor. These microscopic droplet reactors provide a unique microenvironment that facilitates the successful cultivation of a diverse array of microorganisms in a massively parallel fashion, including those that may be recalcitrant to growth on conventional solid media (23). Several remarkable successes have been demonstrated in cultivating previously hard-to-culture microbes, including the identification of previously unknown fluoranthene-degrading *Blastococcus* species from polycyclic aromatic hydrocarbons (PAH) contaminated soil (24) and the isolation of several antibiotic-resistant bacteria from stool samples that grew in droplets and not in Petri dishes (16), thereby expanding the repertoire of cultivable species (25, 26). A recent droplet-based study selectively isolated functional bacteria from the gut microbiome that could utilize metabolites from engineered butyrate-producing bacteria (27, 28). Several isolates, including *Bifidobacterium*, *Bacillus,* and *Lactobacillus*, were obtained, which are deemed beneficial or have probiotic-related properties. Recently, a droplet-based platform was presented for the cultivation of human gut microbiome, showing 2.8 times higher taxa richness compared to batch culture methods (29). Similarly, a single-cell dispensing platform based on droplets was utilized for the isolation of human gut microbiome members with increased efficacy and speed (17). Thus, droplet microfluidics offers a promising solution to overcome some of the limitations of traditional culturing methods.

In this study, we describe a straightforward droplet microfluidic approach for the cultivation, isolation, and analysis of bacterial community dynamics from mouse fecal samples. Our approach encompasses optimized protocols for cultivation under both aerobic and anaerobic conditions within individual droplets, thereby mimicking the diverse microenvironments encountered within the gut ecosystem. Notably, our methodology facilitated the successful isolation of individual colonies originating from individual clonal droplet cultures. Additionally, we extended our investigation beyond microbial isolation to include population-level analysis. By performing 16S rDNA amplicon sequencing of the diverse microbial cultures in droplets, we were able to analyze changes in microbial diversity induced by dietary interventions to the host.

## RESULTS

### Droplet microfluidic workflow

**Fig 1:**
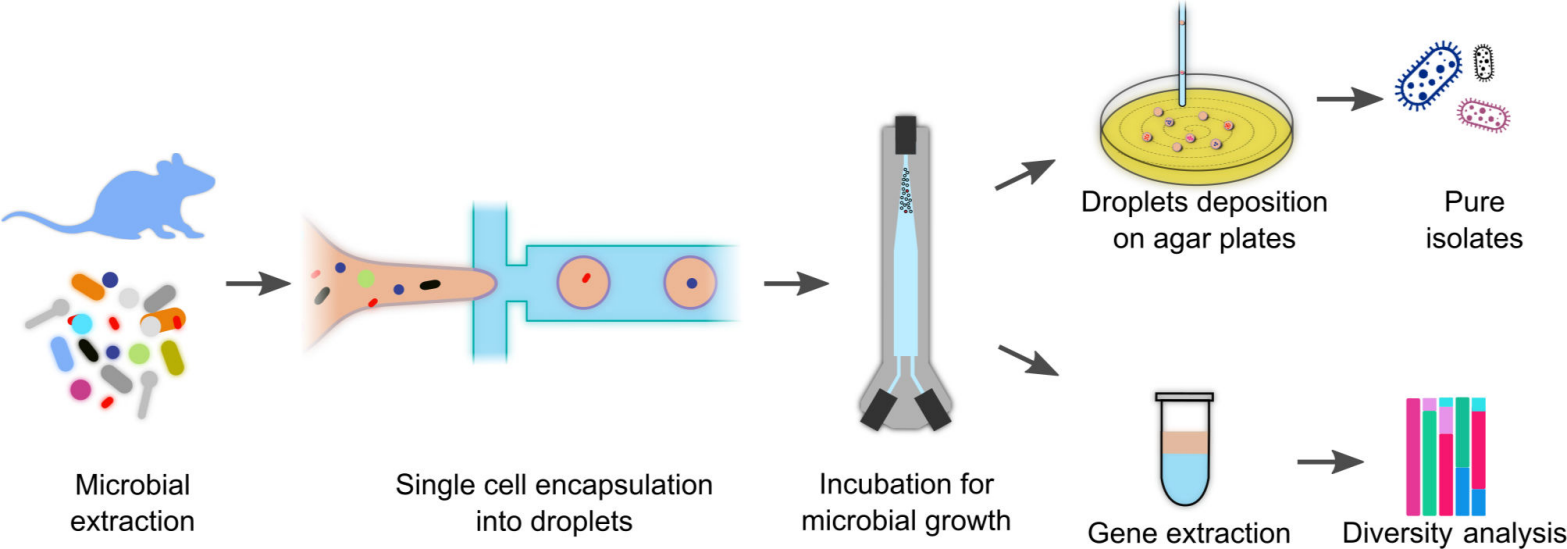
Schematic workflow for cultivation and isolation of microorganisms from mouse fecal sample. Fecal pellets were dissolved in PBS and passed through a 40 µm strainer to retain microorganisms. Droplets were generated from diluted microbial samples targeting single-cell encapsulation and incubated in aerobic and anaerobic conditions. For isolating pure isolates, droplets were individually deposited on agar plates and incubated, resulting in visible colonies, which were identified via Sanger sequencing of their 16S rRNA gene. For community analysis, droplets were then pooled for DNA extraction, followed by Illumina sequencing.

The droplet microfluidic platform employed in this study comprised a streamlined four-step workflow: sample preparation, droplet generation, incubation, and microbial isolation or sequencing (Fig. 1). The initial step involved the extraction of microorganisms from mouse fecal samples, aimed at capturing the diversity of the microorganisms present within the gut ecosystem. Extracted samples were further diluted to reach an inoculation density that guaranteed most of the droplets were inoculated with single cells. We adjusted the average number of cells (lambda, λ) per droplet (volume ~85 pl) to 0.11, resulting theoretically in ~10% of single-cell droplets and less than 0.6% with more than one cell in each droplet following Poisson distribution (30). Nevertheless, the actual occupancy during experiments could vary due to analytical or biological reasons, such as non-viable cells or cell clumping and aggregation, which affect the estimation of the initial cell count.

The second step in the workflow harnessed the power of microfluidics to generate monodisperse droplets at high throughput. A flow-focusing microfluidic chip enabled the controlled formation of picoliter-sized droplets with remarkable uniformity (Fig. S1). In this process, the extracted microbial sample, suspended in an aqueous phase of the cultivation media, was introduced into the central channel of the chip, while a continuous oil phase flowed through the two adjacent channels. At the junction where these three channels converge, the immiscible aqueous and oil phases meet, resulting in the spontaneous generation of discrete droplets encapsulating the microbial cells (31). At a frequency of ~2,000 droplets per second, droplets with a uniform diameter of ~55 µm were generated. A biocompatible surfactant was added to the continuous oil phase to maintain droplet stability and minimize coalescence, ensuring the long-term integrity of these microscopic bioreactors. The resulting droplet population exhibited a very low coefficient of variance (~5%), enabling consistent and reproducible cultivation conditions across individual droplet reactors (Fig. S2).

Following the droplet generation step, the encapsulated microbial cells were subjected to incubation under either aerobic or anaerobic conditions, tailored to accommodate the diverse microbial habitats of the gut microbiome. For aerobic incubation, a specialized dynamic droplet incubator (32) was employed (Fig. 1), with continuous recirculation of the oil phase surrounding the droplets. This setup ensured a homogeneous supply of oxygen to the individual droplet reactors (32). Conversely, anaerobic incubation was achieved by incubating the droplets in tightly sealed glass vials in an incubator within an anaerobic workstation, creating an oxygen-depleted environment conducive to the growth of obligate anaerobes. The droplets exhibited exceptional stability and integrity throughout the incubation period, with minimal or no observable shrinkage in droplet volume (Fig. S2). This remarkable resilience can be attributed to the use of an efficient surfactant, the optimization of incubation conditions, and the gentle handling of droplets throughout the workflow, collectively ensuring the maintenance of a stable microenvironment within each droplet bioreactor. Here, we incubated for only 3 days, but previous work with microbial inocula from environmental samples showed stable droplet cultivation for several weeks (33, 34).

After the incubation phase, the workflow bifurcated into two distinct paths, microbial isolation or community profiling (Fig. 1). For the isolation of individual colonies, the incubated droplets were reinjected into a microfluidic chip, where they were interspaced with an oil phase to maintain a low frequency (~5 Hz). Through integrated capillary tubing, droplets were then deposited on top of agar plates with the help of an XYZ-positioning setup and incubated. The controlled spacing ensured the deposition of well-separated droplets onto agar plates, enabling the growth and isolation of visible colonies originating from the encapsulated clonal microbial cells. Alternatively, for the purpose of diversity analysis of the in-droplet cultivar, 100 µL of the incubated droplets was pooled together and subjected to DNA extraction. The extracted genomic material was subjected to 16S rDNA Illumina amplicon sequencing, providing a comprehensive profiling of the microbial communities cultivated within the droplet microenvironment. To determine if this approach can document the microbiome changes in a mouse experiment, droplet cultivation was performed following specific changes to the mouse diet and analyzed using the community profiling approach.

### Validating aerobic and anaerobic cultivation in droplets

With this workflow established, we first validated the setup and incubation conditions for the prospective cultivation of gut microbes. To this end, we employed two model bacterial strains commonly found in the mouse gut microbiome: a facultative aerobic species, *Enterococcus faecium,* and an obligate anaerobe, *Bacteroides vulgatus*. These strains were encapsulated at a single-cell level and cultivated under controlled aerobic (for *E. faecium*) and anaerobic (for *B. vulgatus*) conditions.

**Fig 2:**
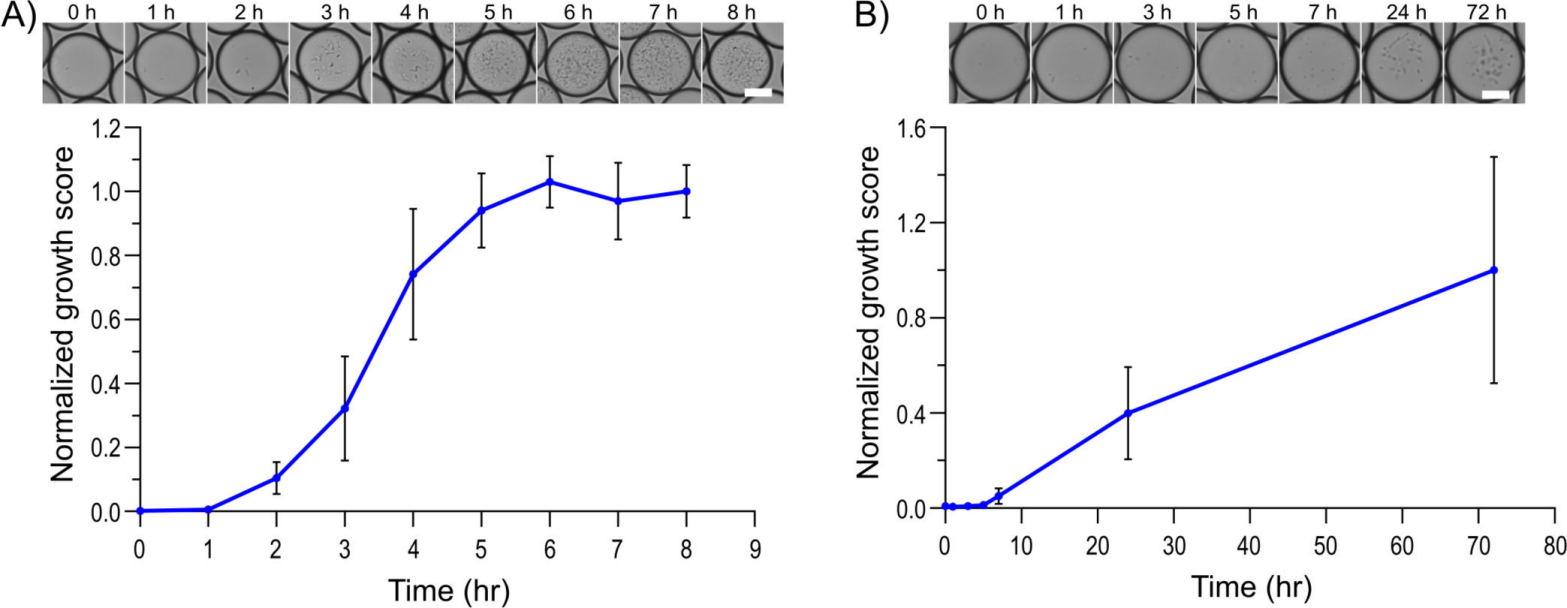
Growth quantification of aerobic and anaerobic strains in droplets. Droplets with single-cell encapsulation of (**A**) *Enterococcus faecium* cultivated in aerobic incubation and (**B**) *Bacteroides vulgatus* cultivated in anaerobic incubation. Droplets were imaged and analyzed for tracking growth via automated image analysis, yielding a normalized growth score. The upper row shows cropped images of a single randomly selected droplet from each measurement time point (see Fig. S3 for full-size images). Scale bars in white color: 25 µm. The lower row shows the growth score normalized to the growth amount at the final time points. For aerobic strain, exponential growth was observed after 2 hours and saturated after 6 hours, while the anaerobic strain had a lower growth rate. Around 3,100 droplets were analyzed for each data point, and error bars represent one standard deviation.

To monitor the progression of microbial growth within the droplet reactors, we employed periodic imaging and automated image analysis (35) throughout the incubation period. Image analysis revealed a significant increase in cell density within the individual droplets over time, a clear indication of successful microbial proliferation under both aerobic and anaerobic conditions (Fig. 2). The encapsulated *Enterococcus faecium* cells thrived in the dynamic, oxygen-rich droplet reactors and reached growth saturation within 6 hours, while the *Bacteroides vulgatus* cells, an obligate anaerobe, exhibited a longer lag phase and slow growth over several days within the anaerobic droplet microenvironment. This validation not only confirms the suitability of the droplet microfluidic platform for cultivating a diverse range of microorganisms but also highlights its versatility in accommodating the distinct metabolic requirements of anaerobic members of the gut microbiome.

### Mouse gut bacteria grow in droplets

**Fig 3:**
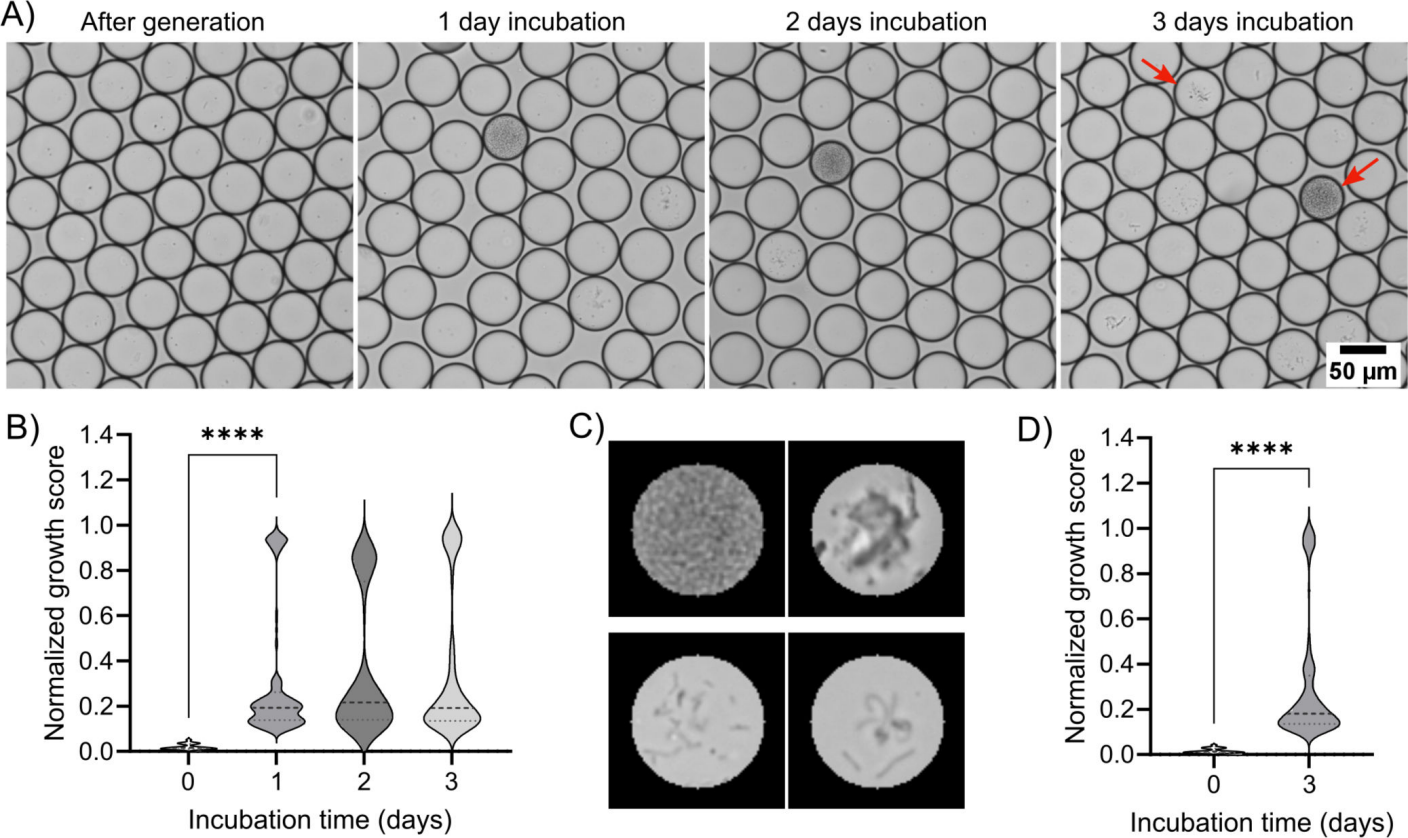
Cultivation and isolation of mouse gut microbial community members using droplet microfluidics. Extracted microorganisms from mouse fecal pellets were encapsulated in droplets and incubated under aerobic and anaerobic conditions. (**A**) Representative images of droplets cultivated under aerobic conditions showing growth of diverse microbial strains (densely filled, clumped, or filaments, marked with red arrows). (**B**) Growth analysis of aerobic droplets showed bivariate distribution after incubation, arising from fast- and slow-growing microorganisms. Significant growth was observed after 1 day of incubation (Mann-Whitney test, *P*-value < 0.0001) and reached saturation after 2 days. Normalized growth score was calculated from non-empty droplets (see Materials and Methods for details). (**C**) Representative cropped images from growth analysis showing droplets with high (upper row) and low (lower row) biomass in droplets. (**D**) Violin plot depicting the normalized growth score of non-empty anaerobic droplets shows significant growth after 3 days of incubation (Mann-Whitney test, *P*-value < 0.0001). For panels **B** and **D**, only non-empty droplets (higher than the growth detection threshold) from a minimum of 5,900 droplets were analyzed for each data set. Median and quartile (Q1 and Q3) values are shown by dashed and dotted lines, respectively.

To capture the gut microbial diversity, we extracted bacteria from fresh mouse fecal pellets, which serve as a representative proxy for the gut microbiome. The extracted microbial suspension was encapsulated into droplets as described above and incubated under both aerobic and anaerobic conditions for 3 days. Images of droplets were taken daily for growth analysis (Fig. 3A). Microorganisms of different sizes, densities, and morphologies were observed, showing their proliferation within the droplets (Fig. S4). The image analysis results confirmed the successful single-cell encapsulation, demonstrating ~95% empty droplets (Fig. S5). In droplets incubated under aerobic conditions, a significant increase in microbial growth was detected after the first day (Fig. 3B), with a bivariate distribution of growth amount, indicating the presence of both fast- and slow-growing species. This rapid proliferation continued, with substantial growth observed on the second and third days. Fast-growing microorganisms resulted in densely filled or clumped biomass in droplets, while only a few cells or filaments were observed in other droplets (Fig. 3C). Similarly, droplets incubated under anaerobic conditions exhibited notable growth after 3 days (Fig. 3D; Fig. S6).

**Fig 4:**
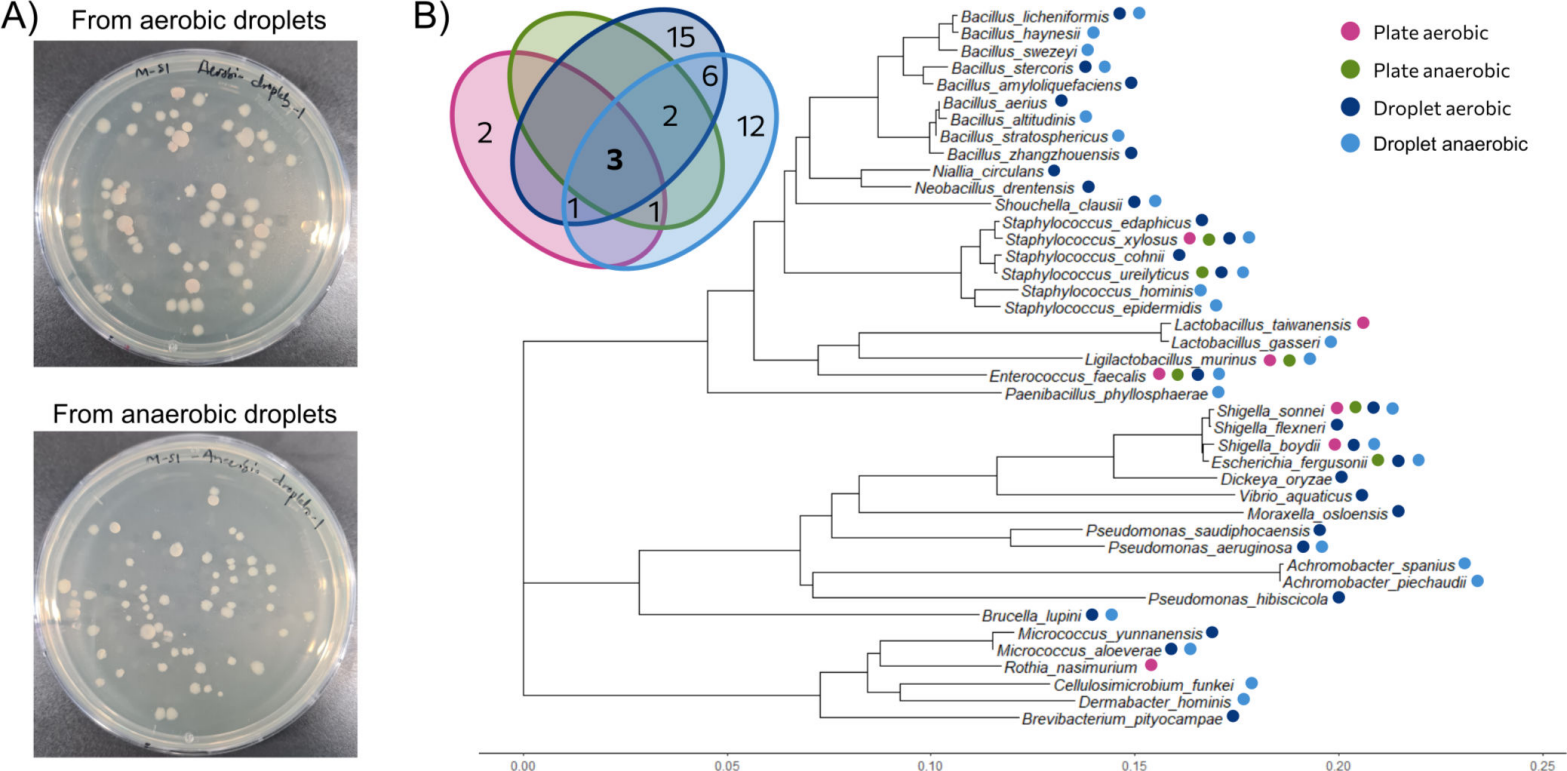
Droplet cultivation extended the cultivable fractions of microbial strains from the mouse gut microbiome. (**A**) Aerobic and anaerobic incubated droplets were dispensed onto agar plates for the recovery and isolation of cultivated microorganisms. After incubation, several colonies of different sizes, morphologies, and colors were observed. (**B**) The maximum likelihood tree shows the unique isolates based on 16S rRNA gene alignment data, from droplet and traditional agar plating cultivation in both aerobic and anaerobic conditions. Only a few pure isolates were obtained from direct agar plating of the fecal extract. Droplet cultivation enriched the library of pure isolates for further analysis. The Venn diagram in the upper left corner shows the number of unique isolates obtained from different cultivation conditions.

After the successful cultivation of gut bacteria within the droplet microenvironment, we proceeded to isolate individual colonies from the incubated droplets. To achieve this, the droplets were dispensed onto agar plates with a capillary-based deposition system (23) and incubated further, facilitating the growth of visible colonies originating from the encapsulated clonal microbial cultures. Remarkably, we observed a diverse array of colonies exhibiting varying sizes, shapes, and colors (Fig. 4A), indicating that conditions in droplets supported the growth of a wide range of species from the fecal microbial community. From both aerobic and anaerobic incubations, several colonies were sub-streaked for culture conservation (Fig. S7).

Pure isolates from both aerobic and anaerobic droplet cultivations were analyzed by Sanger sequencing of the 16S rRNA gene. Forty unique strains were identified, with 12 strains observed in both cultivation conditions (Fig. 4B). Among these isolates, we identified many strains that play crucial roles in maintaining health and balance within the mouse gut ecosystem. Particularly exciting was the isolation of strains with significant probiotic potential, such as *Lactobacillus gasseri* and *Ligilactobacillus murinus* (36–38).

In order to compare the isolation from droplets to a more classical cultivation, we also performed traditional agar plating of the extracted mouse fecal samples onto agar plates with the same medium composition and incubated them under both aerobic and anaerobic conditions (Fig. S8). Several colonies with distinct morphologies were sub-cultured, and DNA was isolated for Sanger sequencing of the 16S rRNA gene. Most strains isolated by direct plating were also observed in droplet cultivations, including *Enterococcus faecalis*, *Shigella sonnei,* and *Staphylococcus xylosus*. Remarkably, the droplet cultivation approach yielded a higher number of unique strains (33 strains from both aerobic and anaerobic conditions) compared to the traditional agar plating method (only two strains) (Fig. 4B).

### Droplets depict changes in bacterial diversity

To gain insights into the composition, diversity, and dynamics of the gut microbial communities cultivated within our droplet microfluidic platform, we used fecal samples from mouse experiments in which animals underwent dietary changes and antibiotic treatment. Fecal samples were collected from mice receiving standard mouse chow (0 days post-feeding [dpf]) and at various time points after supplementation of the drinking water with sucrose (4, 7, 12, and 17 dpf). A second cohort of mice was additionally treated with the antibiotics penicillin, doxycycline, and streptomycin from day 4 onward. As expected (39, 40), mice undergoing antibiotic treatment exhibited a substantial reduction in bacterial growth, resulting in an insufficient DNA yield upon genomic DNA extraction from the raw fecal pellet extracts as well as from droplet cultivations (Fig. S9). Consequently, these samples were excluded from further sequencing analysis.

**Fig 5:**
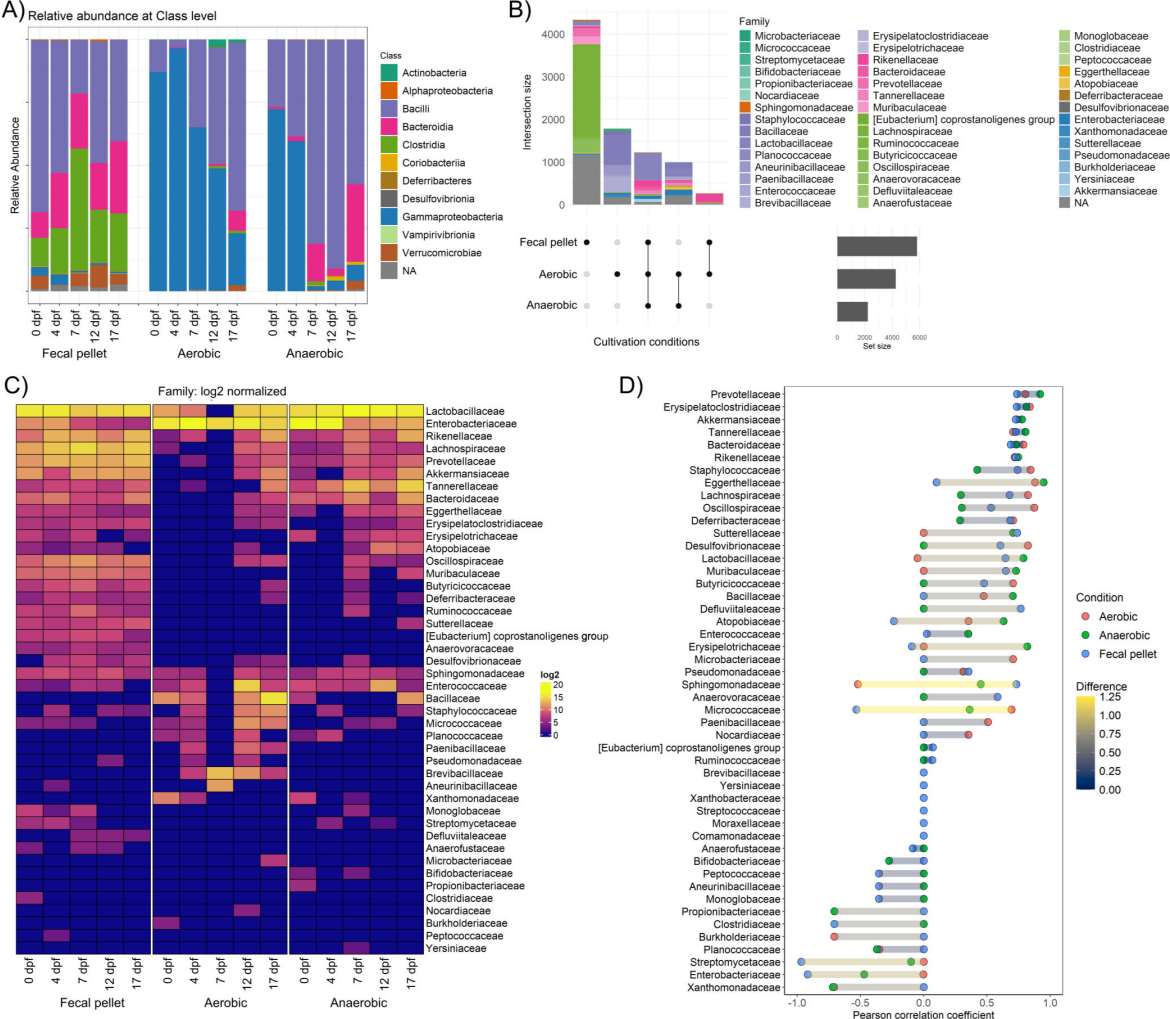
Analysis of bacterial composition in droplet cultivation based on Illumina amplicon sequencing of the 16S rDNA gene. (**A**) Taxonomic composition of microbial communities at the class level. (**B**) Upset plot showing unique and intersecting families across fecal pellet extracts and cultivation conditions. The upper bar plot shows the intersection size of shared taxa, where families are color-coded based on shades of class colors from panel “A.” The lower right bar plot shows the cumulative size of taxa among samples. (**C**) Heatmap showing community structures (displayed at the family level). Intensity of each cell represents the log2-transformed value after normalization of taxa abundances for each sample. (**D**) Pearson correlation coefficients represent changes in bacterial abundance at the family level over time in fecal pellets and droplet cultivations (aerobic and anaerobic). The differences among conditions imply how representative each droplet cultivation is in reflecting changes in abundance within the microbiota. Samples: fecal pellet - droplet populations before incubation (sampled directly after generation, representing composition of fecal pellet extracts); aerobic - droplet populations incubated aerobically; and anaerobic - droplet populations incubated under anaerobic conditions.

With the microbial extract of the fecal samples of mice receiving sucrose, droplets were generated, and 16S rDNA was extracted from droplets directly after generation (labeled as “Fecal pellet”) or after incubating for 3 days in aerobic or anaerobic conditions. A total of 8,596 unique amplicon sequence variants (ASVs) were identified across 15 experimental samples. We observed distinct changes in the composition and diversity of the microbial communities following sucrose supplementation in both the initial fecal sample extract and incubated droplets (Fig. 5A through C; Fig. S10). Alpha-diversity analysis in general indicated a higher level of richness in the original fecal samples compared to all droplet cultivations (Fig. S11).

Out of 8,596 unique ASVs identified from all samples, around half were found only in fecal extracts and the other half in either aerobic or anaerobic droplet cultivations or shared between these conditions and fecal extracts (Fig. 5B; Fig. S12). More than 1,200 ASVs were present in fecal extracts and both droplet cultivation conditions, showing efficient growth and recovery in droplets. These included members of the class *Bacilli*, like *Lactobacillaceae* and *Rickenellaceae*. Surprisingly, many unique ASVs (~2,700) were observed only in droplet cultivations but not in the original fecal pellet extract. This includes many ASVs from families like *Bacillaceae*, *Brevibacillaceae*, and *Aneurinibacillaceae*. Also, it is noteworthy that out of 5,819 unique ASVs detected in fecal pellets, approximately three-fourths were not found in droplet cultivations (Fig. S12). This included highly oxygen-sensitive obligate anaerobes, such as *Lachnospiraceae* and *Oscillospiraceae*, which might have died due to oxygen exposure during fecal pellet handling or droplet generation. In addition, many other taxa belonging to families like *Muribaculaceae*, *Rikenellaceae*, *Anaerovoracaceae*, *Defluviitaleaceae*, and *Anaerofustaceae* were not detected in droplet cultivations, demonstrating that hard-to-culture bacteria in the mouse gut microbiome require a more comprehensive cultivation approach.

Our droplet cultivation approach revealed that certain groups of microorganisms favored growth under aerobic incubation, while others thrived in anaerobic conditions. Distinct clustering of fecal pellet, aerobic, and anaerobic cultivated samples was observed in the NMDS plot (Fig. S13). As expected, facultative anaerobes like *Enterobacteriaceae* and *Pseudomonadaceae*, both members of the phylum *Proteobacteria*, showed higher abundance in aerobic conditions. Similarly, some members of the class *Bacilli*, like *Bacillaceae*, *Paenibacillaceae*, and *Brevibacillaceae*, also had higher abundances in aerobic conditions. On the other hand, aerotolerant anaerobes such as *Lactobacillaceae* and some members of the class *Bacteroidia*, like *Tannerellaceae* and *Bacteroidaceae*, were prominently observed in anaerobic incubation. At the genus level, *Escherichia-Shigella* was the most abundant in aerobic cultivation, and *Lactobacillus* was the most abundant in anaerobic cultivation (Fig. S14).

In that regard, we also designed our study to test whether droplet cultivation can follow and replicate nutrition-induced community changes in the mouse gut. We analyzed the differences in Pearson correlation coefficients for the fecal pellet sample and both droplet cultivation conditions over time (Fig. 5D). Our analysis revealed that the abundance of certain families, such as *Prevotellaceae, Erysipelatoclostridiaceae, Akkermansiaceae*, and *Tannerellaceae*, increased in the fecal pellet sample over time (top part of the plot in Fig. 5D). A similar increasing trend for these families was also observed in both aerobic and anaerobic cultivated droplet samples. Conversely, families like *Enterobacteriaceae, Streptomycetaceae*, and *Clostridiaceae* showed a decrease in abundance in the fecal pellet over time. In general, the trends of increasing or decreasing abundance in droplet cultivations were similar to those observed by direct sequencing of fecal samples for most of the families. However, changes in the DNA abundance of some taxa in fecal samples were only reflected in results obtained from droplets cultivated either aerobically or anaerobically, but not both. In particular, families such as *Atobiaceae, Sphingomonadaceae,* and *Micrococcaceae* exhibited no correlation or even an opposite trend compared to the fecal sample, which can be attributed to the altered incubation conditions and oxygen availability in the droplet system.

## DISCUSSION

We have demonstrated the potential of droplet microfluidics as a powerful platform for the cultivation and isolation of microorganisms from complex microbiomes such as the mouse gut. The droplet-based approach allowed for high-throughput encapsulation of single cells within individual droplets, creating isolated microenvironments that minimized nutrient competition and supported the growth of a diverse array of microorganisms. Our results showed that droplets effectively cultivated both fast-growing and slow-growing species under aerobic and anaerobic conditions and empowered the isolation of a much larger diversity of bacteria compared to conventional plating techniques.

The droplet microfluidic system facilitated the successful isolation of several strains, many of which were challenging to cultivate using traditional agar plate techniques. We were successful in isolating strains with probiotic potential, such as *Lactobacillus gasseri* and *Ligilactobacillus murinus*. Several strains of *Bacillus* and *Pseudomonas* were only observed in droplet cultivations. In contrast, other strains were similarly captured by both droplets and direct plate cultivations. This discrepancy can be attributed to the inherent limitations of conventional culturing techniques, which often favor the growth of fast-growing microbes over slower-growing species due to nutrient competition or an initial preference of the strains regarding liquid or solid media cultivation (16, 23, 41).

While our droplet microfluidic platform has demonstrated remarkable success in cultivating and isolating a diverse array of gut microorganisms, it is important to acknowledge that the isolated strains represent only a fraction of the total microbial community residing within the mouse gut. In our current approach, we employed a single media composition for cultivation, which, although was carefully selected, may not cater to the specific nutritional requirements and growth preferences of all microorganisms inhabiting this complex ecosystem (10, 15, 42). With the integration of multiplexing approaches (35, 43), several media compositions can be simultaneously utilized aiming for the cultivation of a broader spectrum of microorganisms.

Cultivation and isolation of obligate anaerobic strains from fecal samples were generally not very successful via the droplet approach. One possible explanation is the exposure to oxygen during pellet handling and extraction, as well as droplet generation, dispensing, and sub-culturing. To overcome this problem, sample extraction, droplet generation, and dispensing devices could be inserted into an anaerobic chamber for enhanced isolation of anaerobes (17, 29). Furthermore, droplets with detectable growth could be sorted from empty droplet populations based on image (44) or optical (45, 46) analysis, resulting in a smaller pool of selected droplets for dispensing and isolation. This pre-selection would significantly reduce the droplet dispensing time and effort. Additionally, our isolation strategy involved the recovery of microbes from droplets onto solid agar plates, which could potentially limit the growth and colony formation of strains that prefer liquid media environments (23, 47). Deposition of droplets on a microfluidic digital board (48) or on an arrayed plate (49) with the possibility to increase volumes up to nanoliters or microliters could enable a continuous liquid culture environment.

In addition, our current methodology focuses on the individualization of microbes into discrete droplets, which may not be conducive to the growth of certain species that rely on co-culture interactions or synergistic relationships with other microorganisms (11, 15, 50, 51). Encapsulating a small consortium of microbes in droplets (with 2–100 cells/droplet) could maintain the community, enabling microbial pairing and communication within a droplet. It is crucial to recognize that capturing the full landscape of the gut microbiome requires a multifaceted approach, encompassing a diverse array of media compositions, incubation conditions, and co-culture strategies.

Since we are aware of the hurdles for recovering the full spectrum of cultures from droplet cultivations to culture isolates, we also investigated the in-droplet microbial community via sequencing. It is important to note that the droplet cultivation approach for community analysis differs significantly from sequencing-only approaches used to evaluate the mouse gut microbiome, as it only covers viable, growing cells. While this may lead to under-detection of some taxa, it confirms that the detected taxa were present as viable cells in the original sample. Families like *Lactobacillaceae* and *Enterobacteriaceae*, from phylum *Firmicutes* and *Proteobacteria*, respectively, were dominantly present in droplets. This can be attributed to their generalist lifestyle (52) with a capability to thrive in carbohydrate-rich environments in droplets and versatile metabolic capabilities to adapt to various nutrient conditions. Strains from these families, like *Ligilactobacillus murinus*, *Shigella sonnei*, and *Escherichia fergusonii*, were also isolated from dispensed droplets. Additionally, preference for microorganisms from these generalist phyla (*Proteobacteria* and *Firmicutes*) in droplet cultivations was also shown in previous cultivation studies (23, 33).

Differences in growth patterns were observed under aerobic and anaerobic conditions in our droplet cultivation system. As expected (53, 54), oxygen dependency and availability profoundly impacted the composition of the microbial communities in our droplet cultivation approach. Interestingly, cultivation in droplets resulted in the identification of many unique ASVs that were not found in the original fecal pellet extract. This could be either due to their very low abundance in the original fecal sample and supportive growth conditions in the droplets or due to being in a dormant spore form in fecal samples, resulting in inefficient recovery during DNA extraction. It has been described before that certain bacteria transition from a viable state to a dormant spore during passage through the gut sections (55). Likewise, spore formation might have been induced in the fecal exudate due to an aerobic environment. In fact, many ASVs from families like *Bacillaceae, Brevibacillaceae*, and *Aneurinibacillaceae* were observed, which are known for their spore-forming behavior. These low-abundant cells or spores might have grown in droplets to a detectable amount during incubation.

With the change in mouse diet, the temporal shifts in gut bacterial diversity were also reflected in the droplet cultivation system. The trends of increasing or decreasing abundance were similar to those observed in the original fecal samples for most of the families. Additionally, we observed an increase in microbial alpha diversity in droplet cultivations at later time points, whereas the diversity of the fecal extract remained relatively similar. This could be attributed to the presence of diverse low-abundance microbes that are very difficult to recover and their response to the sugar diet, which alters the gut microbiota. We hypothesize that the prolonged continuous sugar feeding benefited microbes that are able to grow on sucrose and enriched the taxa that do not have complicated nutritional requirements, which were therefore easily recovered in droplets. However, this hypothesis needs further verification.

The overall consistency of community trends highlights the capability of the droplet microfluidic system to depict changes in gut microbial diversity, reflecting the dynamic responses of the gut microbiome to dietary interventions (56, 57), even with very different relative and absolute abundances, and regardless of the cultivation bias to be expected from the cultivation media choice and the technique itself. Although the droplet cultivation strategy cannot perfectly imitate the dynamics of microbial communities, we can affirm that droplet cultivation is a technology that allows the detection, monitoring, and investigation of these dynamics. With further advancements in microbial culture recovery from droplets, this strategy might greatly help to bridge the gap from *in vivo* to *in vitro* studies of the relevant microbiome members.

In conclusion, our droplet microfluidic platform provides both a new approach for microbial isolation from complex gut microbiomes and the study of microbiome dynamics. The enhanced and differential isolation of cultivable strains could open the possibility for in-depth studies on their roles in health, disease, and immune functions. Furthermore, 16S rDNA amplicon sequencing revealed that our droplet microfluidic cultivation depicted the temporal changes in microbial community dynamics and diversity induced by dietary interventions, affecting the gut microbiome and opening the door for new experimental designs to investigate microbiome functions upon disturbances.

## MATERIALS AND METHODS

### Bacterial strains and growth media

*Enterococcus faecium* and *Bacteroides vulgatus* cryostocks were streaked on blood agar and incubated under aerobic and anaerobic conditions, respectively. To prepare bacterial suspensions, a single colony of *Enterococcus faecium* was inoculated in Todd Hewitt (THB) liquid media, while Brain Heart Infusion broth was used for *Bacteroides vulgatus*. THB media containing 100 µg/mL Nystatin Dihydrate (Roth) was used for all droplet cultivation experiments from mouse fecal samples. THB agar with Nystatin was used for dispensing, cultivation, and isolation of bacteria from droplets.

### Murine fecal samples

Eight-week-old female specific-pathogen-free C57B/6J mice were purchased from Charles River Laboratories, Sulzfeld, Germany. Mice were housed in the Leibniz Institute for Natural Product Research and Infection Biology-Hans Knöll Institute animal facility. The mice were acclimatized for 1 week prior to the start of the experiment. Mice were divided into control and antibiotic (ABX)-treated groups of five mice per cage. Mice were given a gradient concentration of sucrose in sterile drinking water (2.5% on day 0, 5% on day 1, and 7.5% starting from day two until the end of the experiment [day 17]). For the ABX treatment group, drinking water was supplemented with Penicillin G sodium (1,500 IU/mL, Bela-Pharm, Germany) and streptomycin sulfate (2 mg/mL, Laboratorio Reig Jofre, Spain), and mice were fed irradiated chow containing doxycycline (1,000 mg/kg, Sniff Spezialdiäten, GmbH, Soest, Germany) starting from day 4. Control groups received placebo chow (Sniff Spezialdiäten, GmbH, Soest, Germany). Fresh fecal pellets were collected from individual cages on days 0, 4, 7, 12, and 17 under sterile conditions. Two fecal pellets from each cage were randomly selected and completely homogenized in 1 mL of PBS to prepare a fecal pellet suspension. The suspension was filtered through a 40 µm cell strainer. This suspension was diluted 50-fold in THB medium for droplet generation.

### Design and fabrication of microfluidic devices

Microfluidic chips with a flow-focusing droplet generation nozzle were designed in AutoCAD 2017 (Autodesk) and printed as photomasks (Compugraphics Photomasks). Using photolithography, a mold of SU8-2050 photoresist with ~50 µm channel height was fabricated on a silicon wafer by UV exposure (Kloe UV3) through the photomask following the manufacturer’s protocol (MicroChem). Polydimethylsiloxane (PDMS, Sylgard 184, Dow Corning) replicas were produced following the soft lithography protocol as previously described (58). PDMS chips were plasma bonded (Zepto, Diener) to microscope glass slides, and channels were treated with Trichloro (1H,1H,2H,2H-perfluorooctyl) silane (Sigma). For reinjection chips, a glass capillary was integrated into a chip as previously described (59). For this, one of the outlet ports was punched with a 500 µm biopsy puncher. After bonding, a glass capillary (Polymicro Technologies) was inserted into the chip.

### Droplet generation and incubation

Droplets were generated encapsulating bacterial cells along with growth media using a microfluidic setup (Fig. S15A). Novec oil (HFE7500, 3M) with 2% PicoSurf (Dolomite) was used as a continuous phase. Pressure pumps (Fluigent) were used to actuate fluids into the microfluidic chips with a pressure of ~400 mbar for the aqueous phase and ~600 mbar for the oil phase. Polytetrafluoroethylene tubings, with an inner diameter of 0.75 mm, were used for fluidic connections. A high-speed camera (Pike, Allied Vision, exposure 16 µs, 10 fps) connected to an inverted microscope (Axio Observer Z1, Zeiss, with 5× objective lens in bright-field setting) was used for monitoring the microfluidic operations. Droplets were generated at ~2,000 Hz using a flow-focusing chip with a volume of ~87 pL. Generated droplets were collected either in a dynamic droplet incubator (32) for aerobic incubation (Fig. S15B) or a glass vial for anaerobic incubation. The headspace of the droplet containing the glass vial was purged with nitrogen for 15 min, and the glass vial was transferred to an incubator inside an anaerobic chamber (Coy Labs). Droplets were incubated at 37°C for both conditions.

For the generation of droplets containing *B. vulgatus*, a custom-made glass chamber with a continuous flow of nitrogen was used to house all droplet generation equipment. A high-speed camera (Basler, exposure 20 µs, 10 fps) connected to a modified mini microscope (Bresser) was used inside the chamber for monitoring. A portable pressure pump (Flow EZ, Fluigent) was used for fluid actuation with a pressure of ~400 mbar for the aqueous phase and ~600 mbar for the oil phase. Generated droplets were collected in a glass vial and incubated at 37°C in an incubator inside an anaerobic chamber (Coy Labs).

### Image analysis for growth detection and growth score calculation

Images of stationary droplets at different incubation time points were taken in an observation chamber (Fig. S15C) using an inverted microscope (Axio Observer Z1, Zeiss) and PCO.edge 5.5m camera (PCO) using 10× objective lens. Images from multiple positions were acquired using Zeiss Zen 3.4 software in bright-field channel with an exposure of 2 ms. Image analysis was performed using a Python script as previously described (35). In short, images of individual droplets were processed using Gaussian blur, Canny edge detector, and closing morphology operators. From the final binary images, growth values are determined as the area of white pixels (microbial biomass) divided by the area of the droplet.

The growth score was calculated through the following steps. First, a threshold value was calculated as the mean plus two times the standard deviation of the day 0 droplet measurements, considering non-zero growth values. Droplets with growth were separated by using this threshold value for later time points. The average growth amount (*G_A_*), number (*N*) of these above-threshold droplets, and highest growth amount (*G_A-Max_*) were used to calculate the normalized growth score as follows:


$$Normalized\;growth\;score=\;\frac{G_{A}\times N}{G_{A-Max}},$$

### Droplet dispensing and colony isolation

Droplets were dispensed using a capillary tubing and XYZ-positioning setup (Fig. S15D through F), as previously mentioned (23). After incubation, droplets were reinjected into a reinjection chip using the syringe pump (Nemesys, Cetoni). Novec oil with 0.1% PicoSurf was used for spacing droplets. Droplet frequency was maintained below 5 Hz by adjusting fluid flow rates. Utilizing the integrated capillary tubing, droplets were guided onto agar plates mounted on a positioning device (Cetoni). The positioning device was programmed to move in an expanding spiral pattern, resulting in the deposition of droplets flowing through the capillary tubing. As the spacing oil evaporates, the droplet boundary gets destroyed, yielding bacterial cells on the agar surface. Dispensing on one agar plate took ~6 min. These plates were incubated further at 37°C. Colonies arising from dispensed droplets were picked, sub-streaked, and isolated as pure culture.

### Sanger sequencing and isolate characterization

To characterize isolates, DNA was extracted from pure cultures using QIAmp DNeasy Ultraclean Microbial Kit (Qiagen). PCR was performed to amplify the 16S rDNA gene using standard primer pair, 27F (AGA GTT TGA TCM TGG CTC AG) and 1492R (CGG TTA CCT TGT TAC GAC TT), and PCR Master Mix (Dreamtaq Green, Thermo). The PCR was carried out as follows: pre-denaturation at 95°C for 5 min, 35 cycles of denaturation at 95°C for 30 s, annealing at 55°C for 30 s, elongation at 72°C for 30 s, and final elongation at 72°C for 10 min. Amplified genes were directly used for Sanger sequencing without further cleanup (Eurofins). The resulting sequences with the top species identification match are supplied as supplemental data.

### DNA extraction and quantitative PCR

First sampling was performed immediately after droplet generation, which was marked as “Fecal pellet,” representing the original microbial composition of fecal pellets. After incubation for 3 days, sampling was performed from both cultivation conditions, which were marked as “Aerobic” and “Anaerobic,” respectively. A volume of 100 µL of droplets was sampled at different incubation time points and stored at −20°C until further processing. Once thawed, droplets were broken by using an antistatic gun. DNA was extracted using the DNeasy Ultraclean Microbial Kit (Qiagen) and kept at −20°C until use.

For the analysis of total bacterial abundance, qPCR was performed on the 16S rDNA gene of extracted DNA with Brilliant II Sybr Green qPCR Mastermix (Agilent Technologies) using primers Bac8Fmod (AGA GTT TGA TYM TGG CTC AG) and Bac338Rabc (GCW GCC WCC CGT AGG WGT). The qPCR was carried out as follows: pre-denaturation at 95°C for 10 min, 45 cycles of denaturation at 95°C for 30 s, annealing at 55°C for 30 s, and elongation at 72°C for 25 s. A standard curve was prepared using cloned bacterial 16S rDNA gene, and it was linear from 5 × 10^2^ to 5 × 10^7^, with an *R*^2^ value higher than 0.98. All samples were run in triplicate.

### 16S rDNA amplicon sequencing

The V3-V4 region of the 16S rDNA gene was amplified for Illumina sequencing as previously described (23). Briefly, Q5 polymerase and primers 314F (CCT ACG GGN GGC WGC AG) and 758R (GAC TAC HVG GGT ATC TAA TCC) were used for the amplification of the extracted DNA. The PCR was carried out as follows: pre-denaturation at 98°C for 3 min, 40 cycles of denaturation at 98°C for 30 s, annealing at 55°C for 40 s, elongation at 72°C for 1 min, and final elongation at 72°C for 5 min. After PCR clean-up, amplicons were sequenced on the Illumina MiSeq system by IIT Biotech (Bielefeld, Germany).

### Bioinformatic analysis of sequence data

Illumina amplicon sequence analysis was performed using the DADA2 pipeline for amplicon sequence variants in combination with the DECIPHER package and its specifically formatted SILVA database (version 138) for taxonomic assignment in R/RStudio. The alignment of Sanger amplicon sequences and the construction of the corresponding maximum likelihood tree were performed in MEGA.

### Statistical analysis

GraphPad Prism and R/RStudio were used to make plots and perform subsequent statistical analyses.

## Data Availability

The authors confirm that the data supporting the findings of this study are available within the article and its supplemental material. The individual Sanger sequencing data of isolated bacteria are supplied as supplemental material, and the 16S droplet population sequencing data that support the findings of this study are openly available at NCBI’s Sequence Read Archive database under BioProject accession number PRJNA1273137.

## SUPPLEMENTAL MATERIAL

Data Set S1 aem.00695-25-s0001.xlsx Sanger sequences for all isolated strains from different cultivation conditions.

Supplemental figures aem.00695-25-s0002.docx Figures S1 to S15.

ASM does not own the copyrights to Supplemental Material that may be linked to, or accessed through, an article. The authors have granted ASM a non-exclusive, world-wide license to publish the Supplemental Material files. Please contact the corresponding author directly for reuse.
