# Supplementary material for: A droplet microfluidic strategy for cultivation, investigation, and high-throughput isolation of mouse gut microbiome bacteria
Source: Appl Environ Microbiol. 2025 Jul 10;91(8):e00695-25. doi: 10.1128/aem.00695-25 (PMC12366301; doi:10.1128/aem.00695-25)
Supplement: Supplemental figures — Figures S1 to S15. [file aem.00695-25-s0002.docx]

Supplementary material

A droplet microfluidic strategy for cultivation, investigation, and high-throughput isolation of mouse gut microbiome bacteria

Sundar Hengoju^a^, Ketema Abdissa^a^, Santiago T. Boto^a^, Ashkan Samimi^a^, Karin Martin^a^, Ilse D. Jacobsen^a,b^, Miriam A. Rosenbaum^a,b,c^#

^a^Leibniz Institute for Natural Product Research and Infection Biology – Hans-Knöll-Institute, Jena, Germany
^b^Friedrich Schiller University, Faculty of Biological Sciences, Jena, Germany

^c^Cluster of Excellence Balance of the Microverse, Friedrich Schiller University, Jena, Germany

#Address correspondence to Miriam A. Rosenbaum, [miriam.rosenbaum@leibniz-hki.de](mailto:miriam.rosenbaum@leibniz-hki.de)

*This Supplementary Material contains:*

Figure S1: Microfluidic chip for droplet generation

Figure S2: Comparison of droplet diameter before (blue) and after (red) incubation for confirming droplet stability during incubation

Figure S3: Representative full-size images of droplets at different time points

Figure S4: Selected image of droplets with mouse fecal microbial strains

Figure S5: Growth threshold setting for finding filled droplets

Figure S6: Cultivation of mouse fecal pellet microbial strains in anaerobic condition

Figure S7: Recovery, isolation, and sub-streaking of colonies from dispensed droplets

Figure S8: Colonies obtained from extracted microbial samples from fecal pellets using classical agar plating

Figure S9: Quantitative analysis of total bacterial abundance through 16S rDNA gene quantification by qPCR

Figure S10: 16S rDNA amplicon sequence analysis of microbial composition

Figure S11: Alpha diversity showing population richness for different samples

Figure S12: Venn diagram showing the number of unique ASVs identified in fecal pellet, aerobic, and anaerobic cultivation conditions 1

Figure S13: Box-plot showing difference between aerobic and anaerobic cultivation conditions

Figure S14: Non-metric multidimensional scaling (NMDS) plot showing difference in bacterial diversity due to different cultivation conditions

Figure S15: Images of microfluidic setup

*A separate Supplementary Data file (Excel file) provides the unique 16S rDNA sequences from Sanger sequencing of strain isolates (in relation to Main Figure 4 and Suppl Figures S7 and S8).*


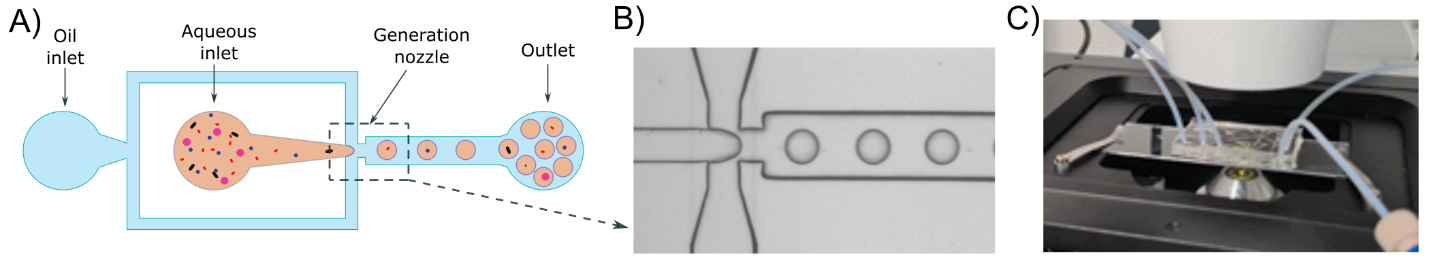


Figure S1: Microfluidic chip for droplet generation. *Left:* Schematic sketch of flow-focusing droplet generation chip. *Center:* Microscopic image at droplet generation nozzle. *Right:* Image of droplet generation chip with fluidic connections on the microscope stage.


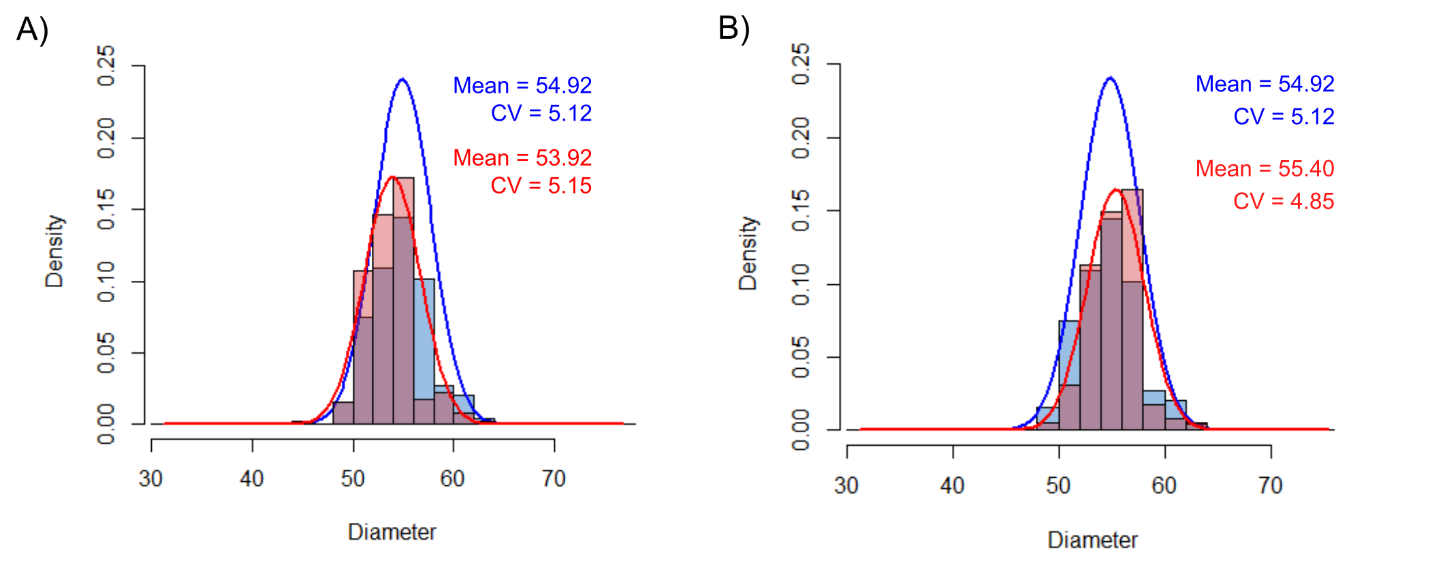


Figure S2: Comparison of droplet diameter before (blue) and after (red) incubation for confirming droplet stability during incubation. *Left:* Droplet incubated in aerobic condition. *Right:* Droplet incubated in anaerobic condition. The unit of diameter on the x-axis is micrometer. More than 3800 droplets were analyzed for each condition.


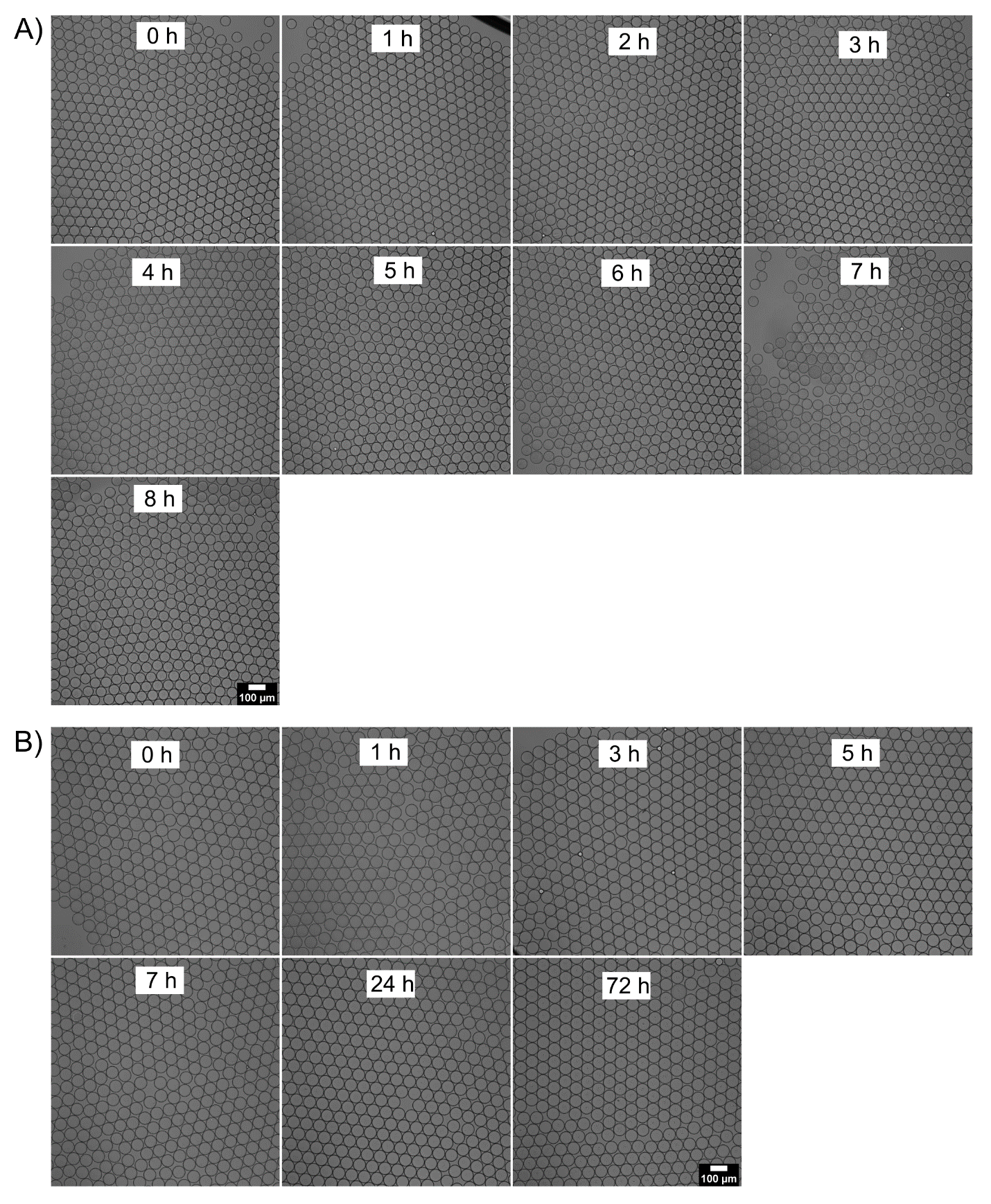


Figure S3: Representative full-size images of droplets at different time points. (a): *Enterococcus faecium* during aerobic incubation in a dynamic droplet incubator. (b): *Bacteroides vulgatus* during anaerobic incubation.


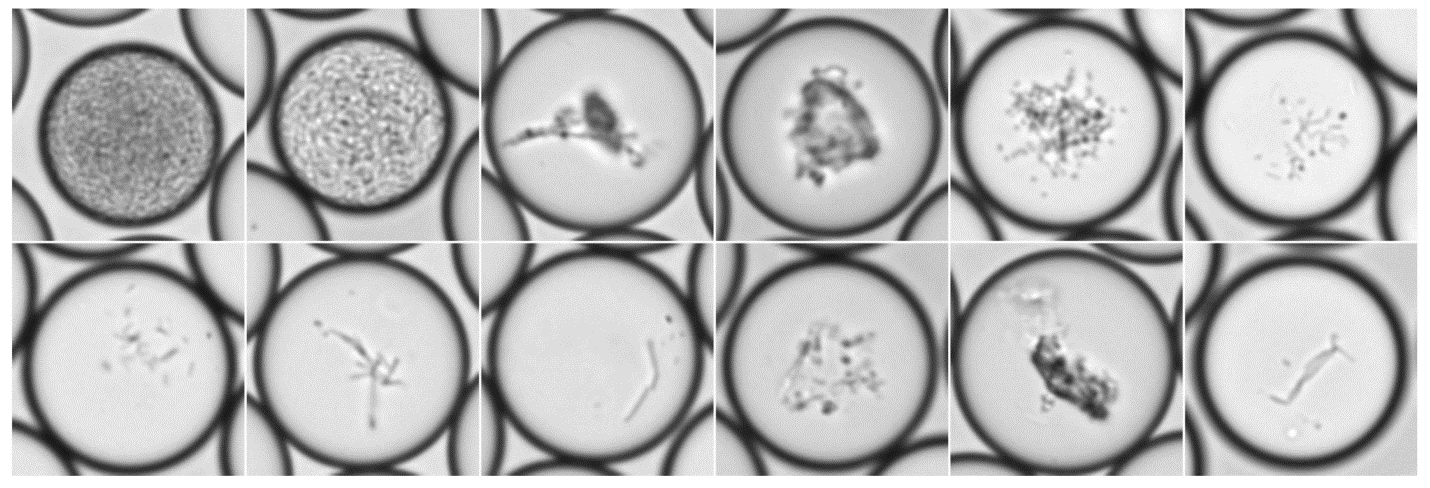


Figure S4: Selected image of droplets with mouse fecal microbial strains after 3 days of aerobic incubation.


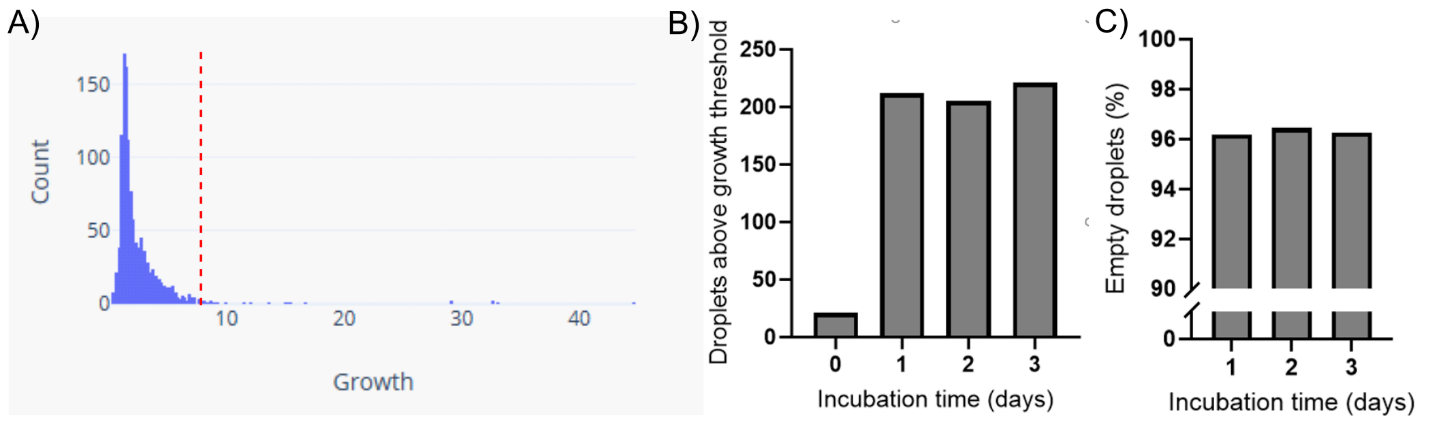


Figure S5: Growth threshold setting for finding filled droplets. *Left:* Threshold was set at mean plus 2 times the standard deviation of bacterial biomass from droplets after generation (red dotted lines at 8.2). *Middle:* Number of occupied droplets detected after thresholding. More than 5900 droplets were analyzed for each dataset. *Right:* Percentage of empty droplets (droplets with detected growth below the threshold line).


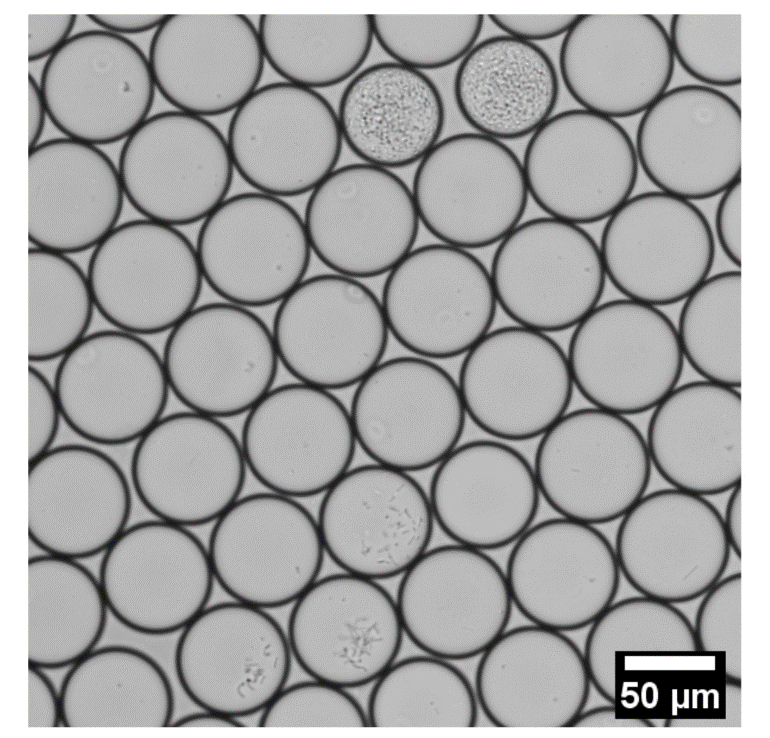


Figure S6: Cultivation of mouse fecal pellet microbial strains in anaerobic condition. Representative image of droplets after incubation for 3 days.


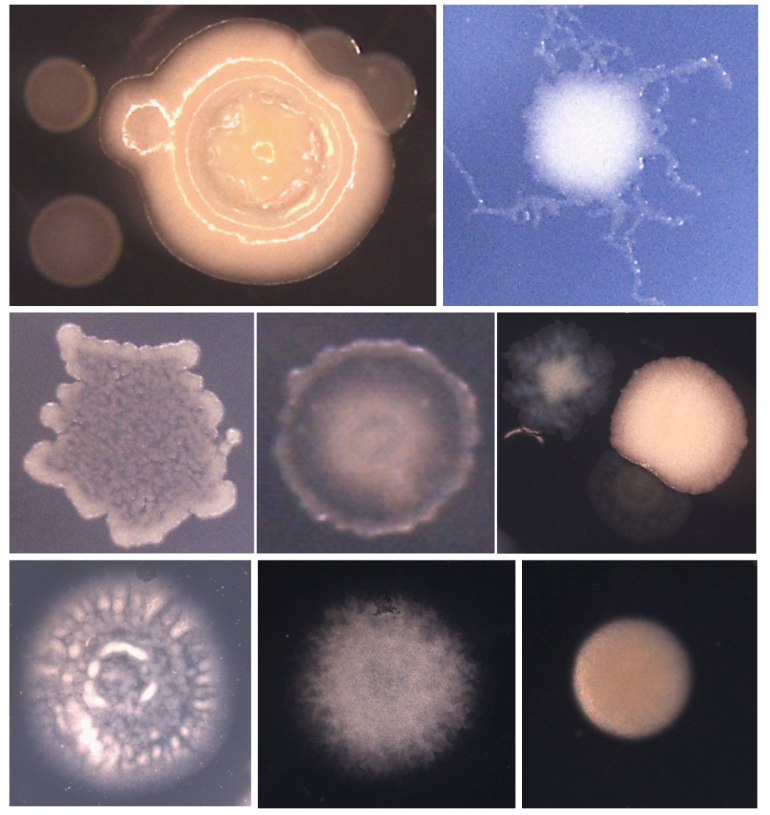


Figure S7: Recovery, isolation, and sub-streaking of colonies from dispensed droplets. Distinct recovered colonies from dispensed droplets were sub-streaked to new agar plates to obtain a pure isolate. Sample images (with different magnifications) of pure colonies showing different morphology after sub-streaking on agar plates.


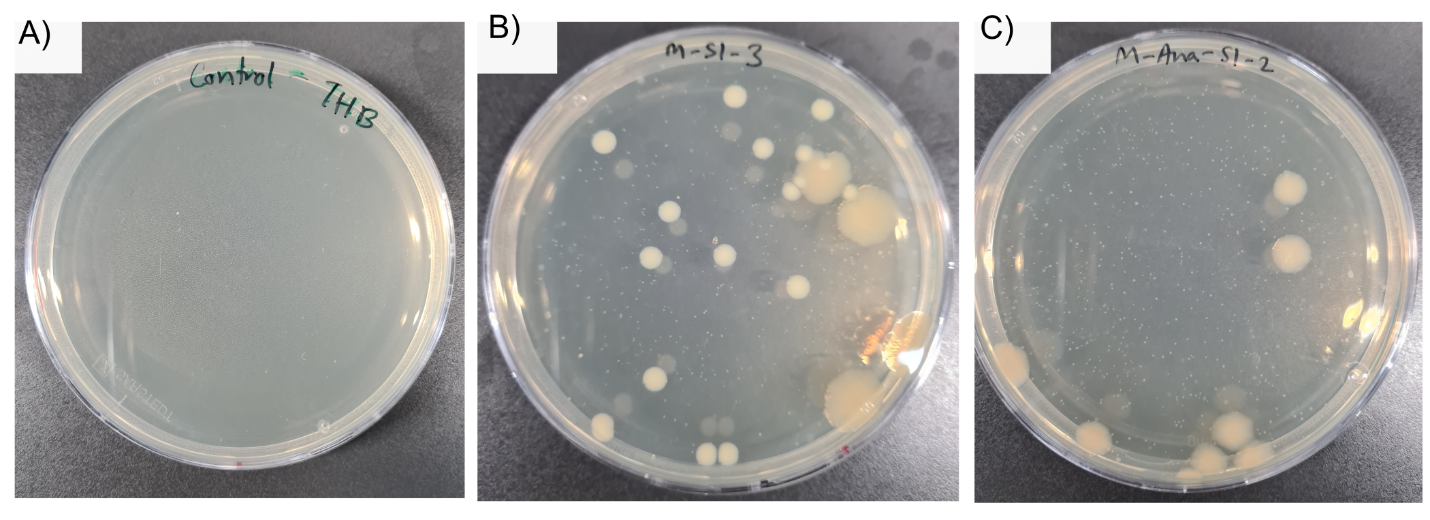


Figure S8: Colonies obtained from extracted microbial samples from fecal pellets using classical agar plating. *Left:* Control agar plate. *Center:* Aerobic incubate plate. *Right:* Anaerobic incubated plate. Only one representative image is shown for each incubation condition out of four plates.


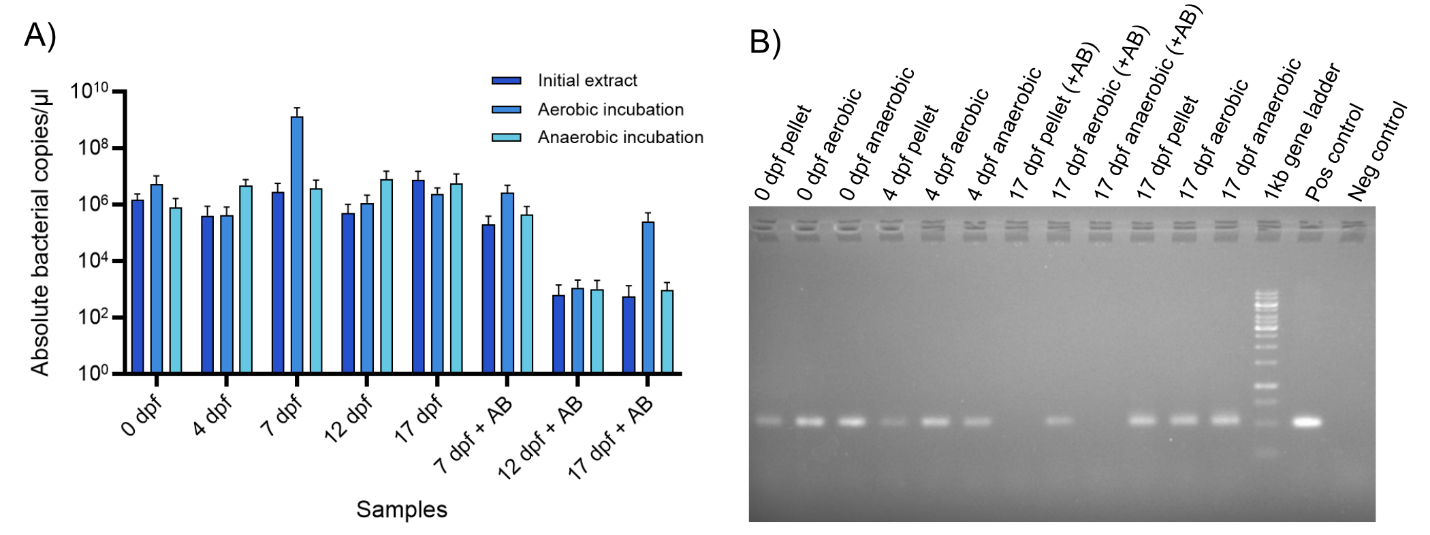


Figure S9: Quantitative analysis of total bacterial abundance through 16S rDNA gene quantification by qPCR. *Left:* Determination of bacterial copies using qPCR. Sugar was co-fed to all mice starting on day 0 (sample for day 0 was collected before sugar co-feeding), and fecal samples were collected and analyzed every 4-5 days for a period of 17 days. Antibiotics (AB) were supplemented with sugar to a subset of mice after day 4. *Right:* Agarose gel image of samples after qPCR.


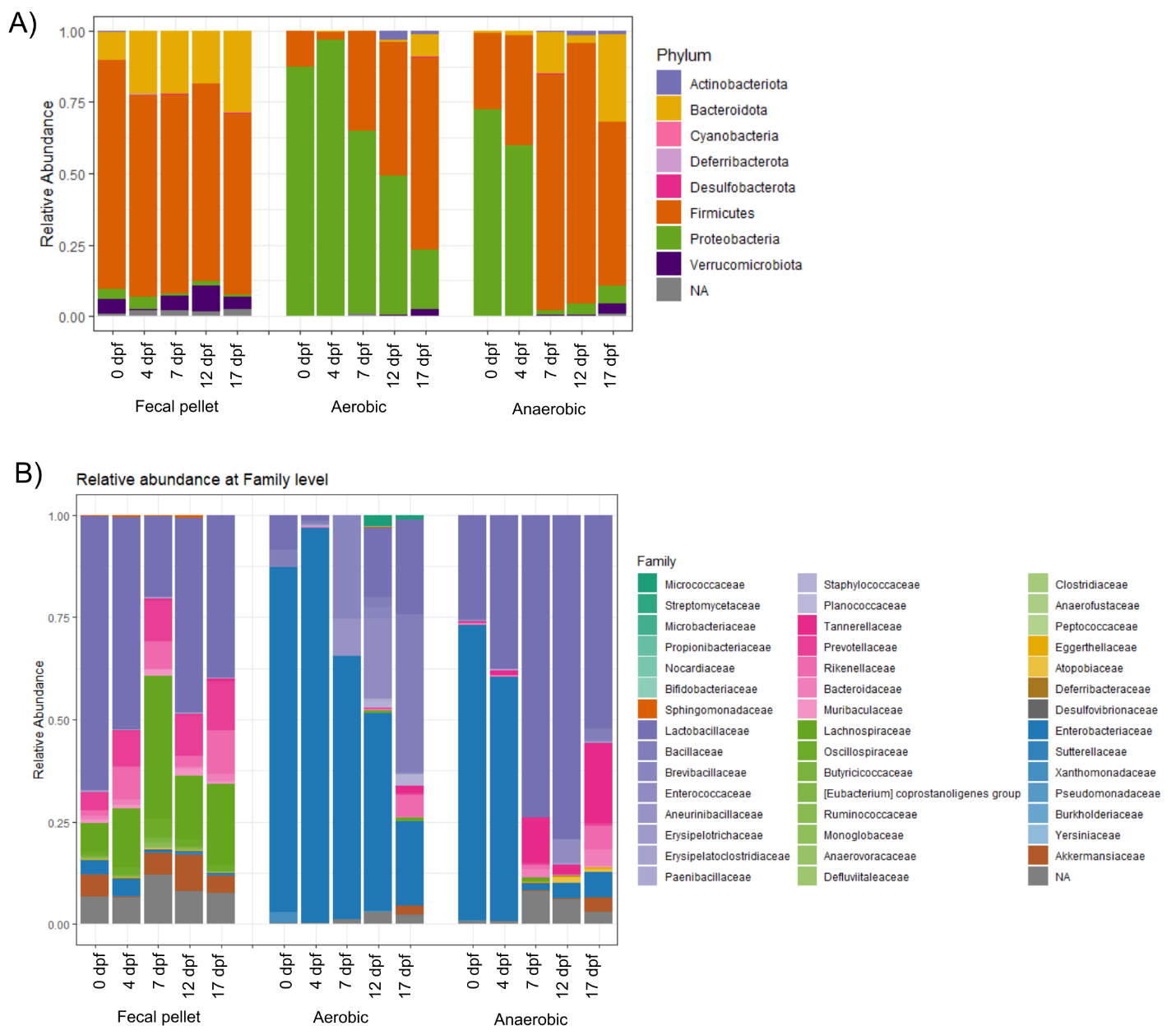


Figure S10: 16S rDNA amplicon sequence analysis of microbial composition. Relative abundance of microbial diversity at Phylum level (a) and family level (b). *Samples*: Fecal pellet: droplet populations before incubation (sampled directly after generation representing the composition of fecal pellets), Aerobic: droplet populations incubated aerobically, and Anaerobic: droplet populations incubated under anaerobic conditions.


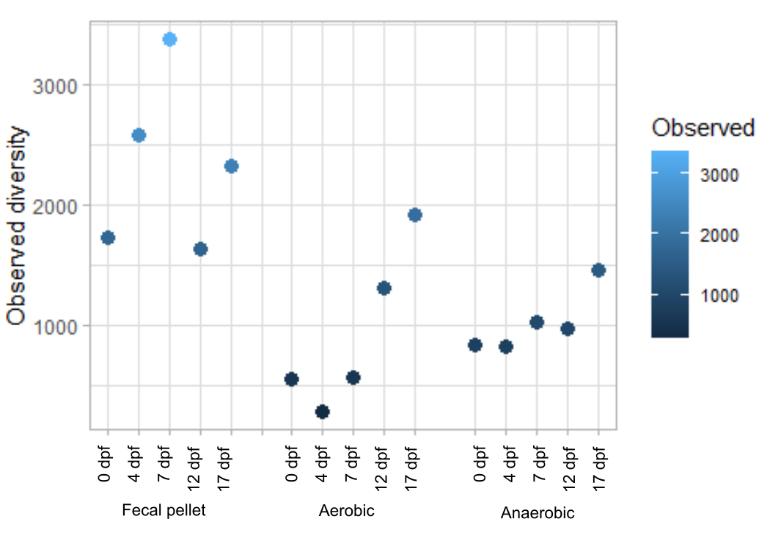


Figure S11: Alpha diversity showing population richness for different samples. Higher the richness in community was observed for fecal pellets. Also, an increase in microbial diversity is observed in droplet cultivations at later time points (12 and 17 dpf). *Samples*: Fecal pellet: droplet populations before incubation (sampled directly after generation representing the composition of fecal pellets), Aerobic: droplet populations incubated aerobically, and Anaerobic: droplet populations incubated under anaerobic conditions.


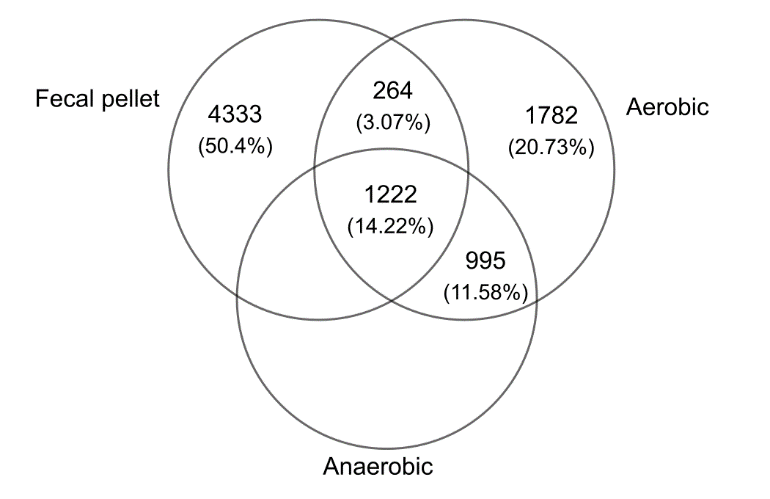


Figure S12: Venn diagram showing the number of unique ASVs identified in fecal pellet, aerobic, and anaerobic cultivation conditions.


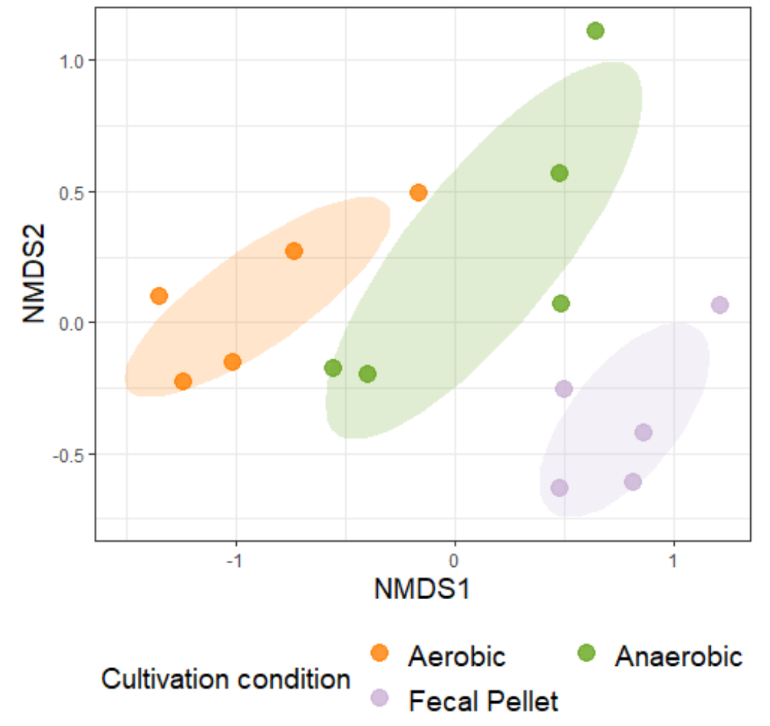


Figure S13: Non-metric multidimensional scaling (NMDS) plot based on the Bray-Curtis dissimilarity method, showing difference in bacterial diversity due to different cultivation conditions. Ellipses of respective sample colors are drawn to highlight distinct clusters.


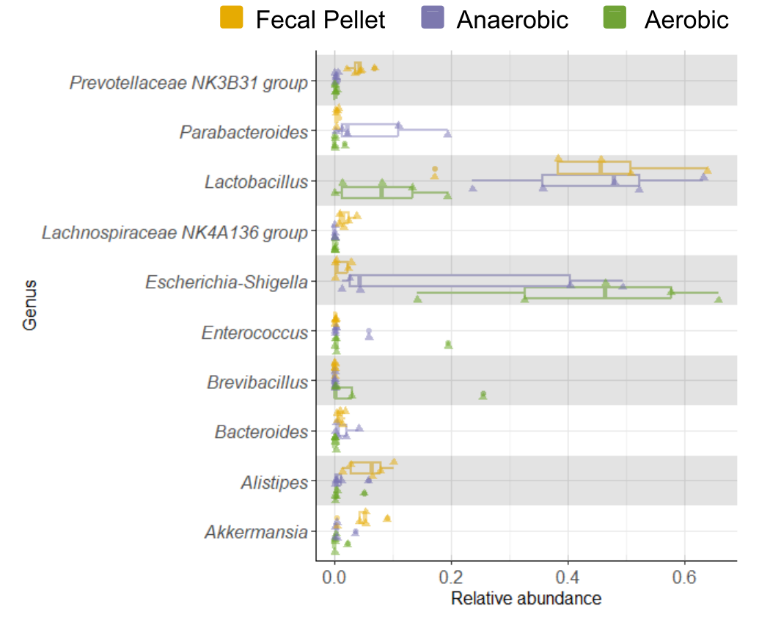


Figure S14: Box-plot showing difference between aerobic and anaerobic cultivation conditions in relative abundances for the top 10 genera. Triangles depict the relative abundances for genera in five samples. *Escherichia-Shigella* and *Prabacteroides* were highly enriched in droplet cultivations, while *Lactobacillus* were maintained in anaerobic droplet cultivation. Fecal pellets, anaerobic and aerobic incubated droplets are shown in gold, blue, and green colors, respectively.


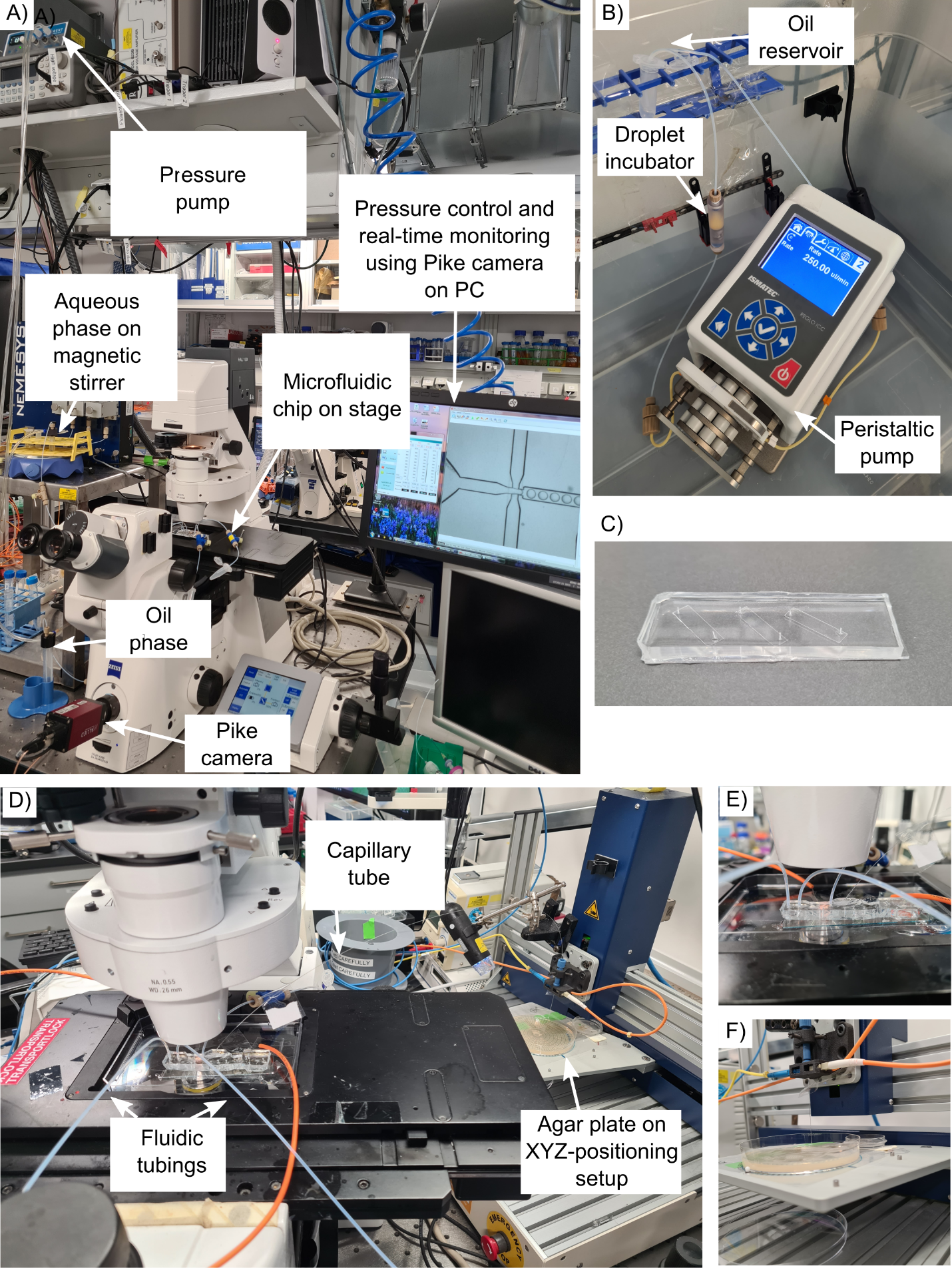
Figure S15: Images of microfluidic setup. (A) Droplet generation setup using microscope (Zeiss), pressure pump (Fluigent) and camera (Pike). (B) Droplet incubation system with 3D-printed droplet incubator & peristaltic pump (Ismatec). Oil (from oil reservoir) circulates into droplet-incubator from top of the incubator to the bottom, with pump at flow rate of 250 µl/min. (C) PDMS observation chamber used for imaging droplets. 3-6 µl of droplets are loaded into each chamber, waited until droplets are stationary, and imaged under microscope (Zeiss). Droplet loaded into observation chamber are discarded after imaging. (D) Droplet reinjection and dispensing setup. Droplets (after incubation) were reinjected into a microfluidic chip integrated with a capillary tubing. A free end of capillary is connected to a XYZ-positioning setup. With defined program, reinjected droplets are dispensed onto agar surface. (E) Image of reinjection chip on a microscope stage, with fluidic tubings for droplets and spacing oil and a capillary tubing. (F) Image of droplet dispensing holder showing capillary tube and agar plate on a holder.
